# Patterns of blood biochemical parameters of peripartum dairy cows raised in either smallholder or semi-commercial dairy farms in Thailand

**DOI:** 10.14202/vetworld.2021.649-655

**Published:** 2021-03-17

**Authors:** Supawit Triwutanon, Theera Rukkwamsuk

**Affiliations:** Department of Large Animal and Wildlife Clinical Sciences, Faculty of Veterinary Medicine, Kasetsart University, Kamphaeng Saen, Nakhon Pathom 73140, Thailand

**Keywords:** β-hydroxybutyrate, negative energy balance, non-esterified fatty acid, smallholder dairy farm, tropical area

## Abstract

**Background and Aim:**

Data about dynamics of serum biochemical parameters and negative energy balance (NEB) related conditions differ among geographical areas or farm types. It should be cautious about applying those data to justify control and prevention strategies of NEB problems. Therefore, dynamics of blood biochemical parameters related to NEB condition of peripartum dairy cows raised in either smallholder (SH) or semi-commercial (SC) farms were studied.

**Materials and Methods:**

Thirty-two healthy pregnant dry cows were selected from five dairy farms in Western part of Thailand, including 15 and 17 cows from three SH and two SC farms, respectively. Blood samples were collected at 2 weeks before the expected calving date, and 1, 2, 4, and 8 weeks postpartum to determine concentrations of glucose, non-esterified fatty acid (NEFA), and b-hydroxybutyrate (BHBA). Body condition scores (BCSs) and milk yields were also recorded.

**Results:**

Dairy cows in both farm types suffered from NEB by elevation of serum NEFA and BHBA, and loss of BCS postpartum. Degrees of elevation in serum NEFA and BHBA were different between farm types. The SC cows showed more BCS loss postpartum, but lower serum NEFA. In the SH cows, even with less BCS loss, the cows showed high serum NEFA concentrations.

**Conclusion:**

Dairy cows in our study entered NEB condition even with low milk yields. Moreover, elevation of serum NEFA and BHBA postpartum was higher in our studied cows as compared with other studies in high producing cows of commercial dairy farms in temperate areas.

## Introduction

Dairy cow production in tropical areas includes Central America, South America, tropical Asia, and Saharan Africa [1]. In these areas, dairy cattle farms are dominantly smallholder (SH) type, in which the breed of dairy cows is mainly dual-purpose or crossbred. In Thailand, dairy cattle industry has been developed since 1960. Genetic improvements of Thai dairy cows were done using imported semen, embryo, and live animals by the Department of Livestock Development (DLD) under the Tropical Holstein Dairy Cattle (Master Bull) project to provide better adaptation to humid-hot climate and to overall farming managements in tropical areas. Genomic study on fertility and milk production of Thai tropical Holstein dairy cows was conducted by Konkruea *et al*. [2]. For Thai Holstein dairy cows, milk production is relatively lower when compared with those cows in temperate areas. The other concerning factor in tropical dairy cows is heat stress, which directly or indirectly affects dry matter intake (DMI) and generates stress to the cows [3]. For milking propose SH farms, the farm types are classified by crop-livestock system with the use of agricultural plant by-products such as rice, cassava, and palm oil. Not only the agricultural by-products but tropical forages for dairy cows also provide lower quality and nutrients compared to most forage in temperate zone [4]. All these factors cause a reduction of DMI together with an inability of smallholding farmers to successfully provide proper nutritional management to dairy cows, especially during transitional period, possibly leading to negative energy balance (NEB) condition and related consequences.

NEB condition is one of the major concerning conditions of dairy cow production system. Failure of nutritional management during peripartum period results in NEB condition that potentially alters metabolic homeostasis of the cows. In NEB condition, decreased serum glucose, increased lipolysis activity, elevated serum non-esterified fatty acid (NEFA), and b-hydroxybutyrate (BHBA) occur. Severe condition results in subclinical ketosis and fatty liver disease that have negative effects on milk production, reproductive performance, and health condition throughout lactation period [5,6]. Severity of NEB condition depends on various factors related to nutrient demand and nutrient intake, including milk yield, breed, heat stress condition, quality of feed, and nutritional management system [7]. Tropical dairy farms are unique in all mentioned factors compared to temperate dairy farms. Although there are several studies on tropical dairy cattle farms, most of them have been kept under commercial farming system (medium- to large-scale farming type). Considering differences in many factors, those studied results did not reflect the main population of tropical dairy farms, which are usually SH type (Vietnam 6.9 cattle/farm, Thailand 20.1 cattle/farm, India 1.8 cattle/farm, and Pakistan 1.5 cattle/farm; [8]). A study of SH dairy farms in tropical area demonstrated dynamics of blood biochemical parameters, including high serum NEFA and triacylglycerol (TAG) concentrations at 2 weeks before the expected calving date [9]. The use of data about dynamics of serum biochemical parameters and NEB-related condition from different geographical areas or farm types could mislead control and prevention strategies of NEB problem.

This study aimed to determine differences in the dynamic pattern of blood biochemical parameters of peripartum dairy cow raised by either SH or semi-commercial (SC) scale farmers in Thailand.

## Materials and Methods

### Ethical approval

All procedures related to use of animals were approved by Kasetsart University’s Institutional Animal Care and Use Committee (ACKU62-VET-098).

### Location, animals, period, and farms

The study was carried out in five dairy farms located in central part of Thailand (15°00’0.00” N 100°00’0.00” E). Types of farming were classified into SH and SC groups by herd size, nutritional management, and milk yield (Table-1). All farms were registered in Thai DLD system with the use of tropical Holstein frozen semen as selected sire for artificial insemination.

**Table-1:** Classification of farm type.

	Smallholder (SH)	Semi-commercial (SC)
Milking cows (cows)	<20	>20
Milk yield (kg/day)	<13	>13
Nutritional management	Forage and non-forage fiber base diet, none production-based diet feeding	Balance forage and concentrated diet, production-based diet feeding
Number of farms	3	2

In total, 32 healthy pregnant dry cows (crossbred Holstein Friesian, 15 cows in SH and 17 cows in SC) were recruited for the study from October 2017 to February 2018. The forages used in these farms were varied according to their availability, including rice straw, by-product corn (baby corn; *Zea mays var. rugosa*) silage without husk, cassava ethanol by-product, cassava starch by-product, cassava pulp, and pineapple peel. Concentrates were commercial feed topping with dry cassava or soybeans meal in some farms. In SH farm, there was no gradual increase of concentrates for adaptation of rumen function and no adjustment of the amounts of concentrates fed to the cows based on milk production; and the diets were mainly roughage and non-forage fiber. In SC farm, there was a gradual increase of concentrates feeding for adaptation of rumen as well as an adjustment of the amounts of concentrates based on milk production. The diets were balanced between roughages and concentrates. Housing management systems for the SH group were tied stall (one farm) and free stall (two farms) barn, and for the SC group were free stalls (two farms). Although the SH group might be heterogeneous in terms of keeping animals (tie-stall vs. free-stall), these three farms were matched by most within-farm management as much as possible.

### Sample collections and records

Blood samples were collected by venipuncture from the coccygeal vein from all cows at 2 weeks before the expected calving date and at 1, 2, 4, and 8 weeks postpartum. After collection, all blood samples were left atroom temperature (28°C) for 30 min before centrifugation at 1200× *g*; thereafter, serum samples were harvested and stored at −20°C until determination of the concentrations of glucose, NEFA, and BHBA. Body condition scores (BCSs) and milk yields of all cows were recorded at the time of sample collection. Sample collection, milk yield record, and BCS assessment were performed by only one well-trained veterinarian.

### Sample analysis

Serum concentrations of glucose (Glucose, Erba Lachema S.R.O., Karásek 1d, 621 00 Brno, Czech Republic), BHBA (Ranbut, Randox Laboratories Ltd., County Antrim, UK), and NEFA (NEFA, Randox Laboratories, Ltd., County Antrim, UK) were measured using the commercially available test kits as indicated.

### Statistical analysis

All data were explored for their normality using Shapiro–Wilk test. Normally distributed data were subjected to analysis of variance using sampling days as repeated measures. Comparison of blood glucose, NEFA, and BHBA concentrations and BCS data were performed using paired t-test. Non-parametric analysis of repeated measures analysis of variance and paired t-test, where appropriate, were performed using Friedman test and Mann–Whitney U-test, respectively. The significance was preset at p<0.05.

## Results

**Figure-1:**
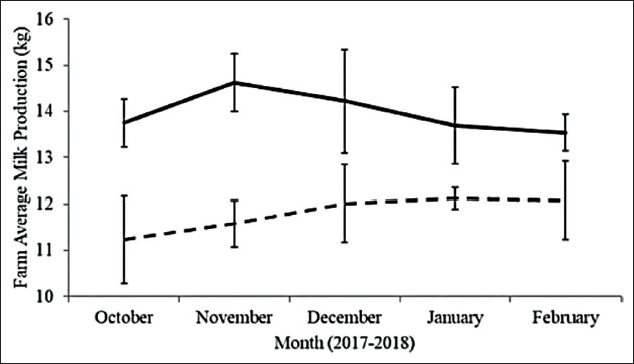
Farm average milk productions in smallholder (broken line, n=3) and semi-commercial (solid line, n=2) during experimental period, error bars stand for standard deviation.

The monthly farm average milk production of both farm types is shown in Figure-1. Milk yields of each group of farm type (SH vs. SC) were calculated as farm average milk production using amount of milk production per farm (kg) and total milking cows. Average milk production throughout the experimental period was 11.80±0.39 and 13.97±0.45 kg/milking cow/day for SH and SC farms, respectively. The milk yields of dairy cows from SC farms were higher than those from SH farms.

**Figure-2:**
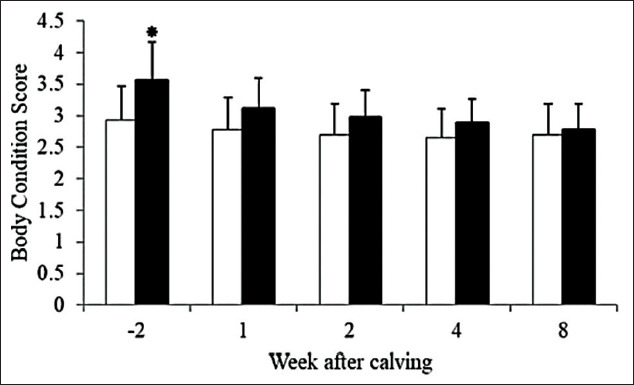
Dynamics of body condition score at 2 weeks before expect calving date at 2 weeks before parturition and 1, 2, 4, and 8 weeks after parturition of smallholder (□, n=15) and semi-commercial (■, n=17), error bars stand for standard deviation.

Average BCS at 2 weeks before the expected calving date and 1, 2, 4, and 8 weeks after parturition of SH and SC is shown in Figure-2. There were significant changes in dynamics of average BCS in SH (p=0.006) and SC (p<0.001). Average BCS at 2 weeks before the expected calving date was lower for SH cows than for SC cows (p=0.005). For both farm types, dairy cows showed a decline in BCS throughout postpartum period.

**Figure-3:**
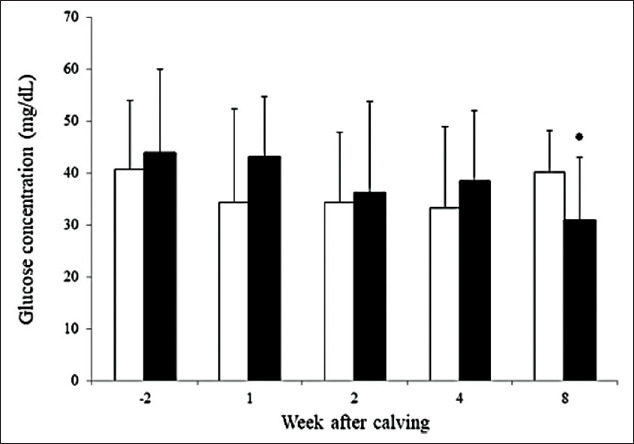
Dynamics of serum glucose concentration at 2 weeks before expect calving date and 1, 2, 4, and 8 weeks after parturition of smallholder (□, n=15) and semi-commercial (■, n=17), error bars stand for standard deviation.

Dynamics of serum glucose concentrations for both farm types are shown in Figure-3. There were significant changes in dynamics of average serum glucose concentrations of SC cows (p=0.022). Average serum glucose concentrations at 8 weeks postpartum for SH cows were higher than for SC cows.

**Figure-4:**
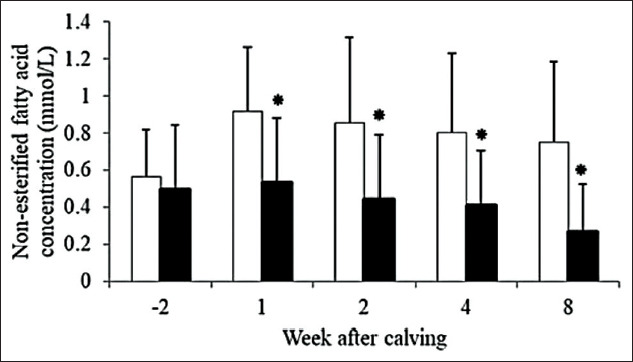
Dynamics of serum non-esterified fatty acid concentration at 2 weeks before expect calving date and 1, 2, 4, and 8 weeks after parturition of smallholder (□, n=15) and semi-commercial (■, n=17), error bars stand for standard deviation.

Serum NEFA concentrations are presented in Figure-4. Average serum NEFA concentrations at 1, 2, 4, and 8 weeks after parturition were higher for SH cows than for SC cows. There were significant changes in dynamics of average NEFA of SH (p=0.004) cows. Patterns of NEFA during peripartum period were similar in both groups, which were peak at 1 week postpartum and gradually decreased by time, and differed in the degree of changes.

**Figure-5:**
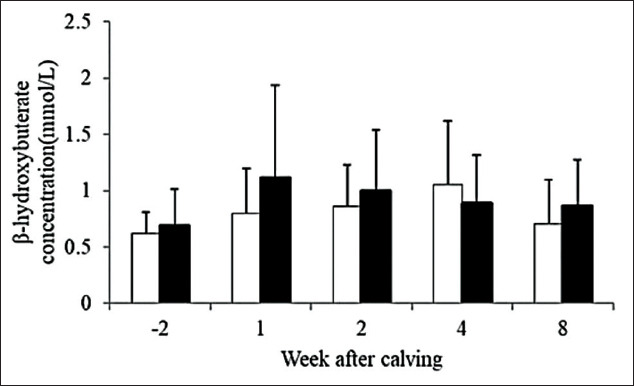
Dynamics of serum β-hydroxybutyrate concentration at 2 weeks before expect calving date and 1, 2, 4, and 8 weeks after parturition of smallholder (□, n=15) and semi-commercial (■, n=17), error bars stand for standard deviation.

Serum BHBA concentrations are presented in Figure-5. For serum BHBA concentrations, there were significant changes in dynamics of average BHBA of SH (p=0.011) cows and SC (p=0.002) cows. Cows from both farm types had the lowest average concentration before parturition and gradually increased postpartum. Peaks of average BHBA concentration were different, which were observed at 4 and 1 weeks postpartum in SH and SC, respectively.

## Discussion

For milk production, it was not possible to measure individual daily milk production due to farm limitations. However, data of farm average milk production were used in this study. Overall data about daily milk yield in Thailand were 12.17 kg/cow/day, calculated from 3.945±1.537 kg of milk/lactation; and a 324±97 days in lactation length were reported in the previous study [10]. Compared with other tropical Asian countries, daily milk production in our study was 3 kg/cow/day higher than in Pakistan [11], 3.78 kg/cow/day higher than in India [12], and 7.28 kg/cow/day higher than in Malaysia [13].

Within this study, farm average milk yield in the SH group was lower than in the SC group, which was correlated to farm overall practices and management systems (more intensive management in SC compared with SH). Medium- to large-scale dairy farms, which were classified as SC farms in this study, had similar farm practices to that recommended for the dairy farms in developed countries. SC type of farm was considered as only minor type of farming in Thailand and in tropical countries. Another limitation of milk production data in our study was that it was unable to define days in milk of all cows in each farm; therefore, differences in milk production might result from lactation phases.

Prepartum BCS was normally recommended at 3.25-3.5 in a common dairy farm practice [14]. Data from our study showed that BCS of dairy cows in SH farms was lower than the recommended level. Change in BCS postpartum was a major point for monitoring NEB condition in dairy cows [15] that should not lose more than 0.5 units of BCS postpartum. In our study, losses of BCS were 0.28 and 0.67 in SH and SC (2 weeks before the expected calving date to 4 weeks postpartum), respectively. When considered by loss of BCS, SC was in NEB stage. In the SH group, though loss of BCS was lower than SC, the BCS was still in the range of recommended level; and the amount of milk yields was greatly lower than commercial farms. Moreover, other serum biochemical parameters should be concerned for evaluated NEB condition in the SH group.

Differences in BCS loss between the two farm types could be resulted from many factors, including milk production, nutrition, and farm management. Compared with other studies in tropical dairy farms, postpartum BCSs were reported to start a gradual increase at 3 weeks postpartum [16], which were observed in SC cows. In contrast to SH cows, the BCS was still continually declined through 8 weeks postpartum, which might be caused by failures in nutritional management of SH dairy farms. BCSs in tropical prepartum cows were reported [9,17], which were lower than expected BCS of 3.5. Loss of BCS postpartum in SH farms in this study was in agreement with various studies that reported the BCS loss ranging from 0.19 to 0.49 in tropical dairy cows with approximately 15 kg/day of milk production in early lactation period [9,18]. The SH group showed less reduction of BCS postpartum compared with the SC group. The SC cows lose BCS as same as other studies [19,20] that found 0.7-1.0 unit loss in 31.15 kg/day of milk production cows in temperate country, which was much higher than our study.

Dynamics of serum glucose concentrations were also related to Kaewlamun’s study [16] that found an immediate decrease postpartum in the tropics. Degree of decreasing in serum glucose concentrations (2 weeks before the expected calving date to 2 weeks postpartum) was 15% and 17% in the SH and SC groups, respectively. Compared with higher percentage (26%) of decreasing in serum glucose from the previous study [9], the different results could be due to milk production and nutritional management. And, the variation in serum glucose in our study might be due to stress or differences in sampling times after feeding. Difference in serum glucose concentration was lower in SC as compared to SH group.

Change in serum NEFA was in contrast to the study by Rukkwamsuk *et al*. [17] that found non-significant differences between prepartum and postpartum concentrations. However, in agreement with the previous study by Chankrachang and Hongyantrachai [18], the NEFA concentrations differed between prepartum and postpartum. Dynamic patterns were similar to the previous studies in tropical cows [16] that found NEFA concentration highest at 1 week postpartum and tended to decrease by times. Percentage of increase in serum NEFA between prepartum and postpartum was 51% and −10% in the SH and SC groups, respectively, which was considered greatly higher than the previous study that showed only 13% of increase [20]. Average serum NEFA concentrations at 2 weeks prepartum in our study (0.56±0.26 and 0.49±0.35 mmol/L for SH and SC, respectively) were classified as NEB condition when using the criteria from high producing temperate country [21]. Postpartum serum NEFA concentrations in SC cows were correlated with the previous study by Rukkwamsuk [17]. Higher NEFA concentrations in postpartum period suggested that there was more lipolysis activity [22] in the SH group even though SH cows had less fat storage, BCS, and milk production.

Prepartum serum BHBA concentrations were lower when compared with the study of Whitaker *et al*. [23] but higher than the other study by Rukkwamsuk *et al*. [24]. Interestingly, peaks of serum BHBA concentrations postpartum were at week 1 postpartum in SC which were agreed with the study by Whitaker *et al*. [23]. Peak of serum BHBA concentration occurred lately in SH, reaching highest concentrations at weeks 4 postpartum.

Classification of NEB using serum NEFA and BHBA concentrations was reviewed in the previous study [21]. The prevalence of NEB classified by various biochemical parameters in our study is shown in Table-2. Compared to other studies in the tropics, our study showed higher in the prevalence of NEB at 33% prepartum (BHBA >0.6 mmol/L), 33% (BHBA >1.0 mmol/L; [23]), and 9% postpartum (BHBA >1.2 mmol/L; [10]). From serum concentrations of biochemical parameters together with percentage of elevation in serum BHBA and NEFA above baseline values, there were NEB condition occurred in both groups of peripartum dairy cows. NEB was considered as a multifactor condition, but one among those important factors was milk production. Normally, tropical areas, including Thailand had lower quantity of milk production compared to temperate areas. Severity of farmers’ mismanagement in combination with heat stress and other environmental factors [24] could result in reduced feed intake, as a consequence, NEB was developed, which also found in our study. When considered only within country milk yields, SH cows that had lower milk yields than the country average showed high degree of NEB condition as compared to SC cows that had higher milk yield.

**Table-2:** Percentage of cows classified by serum non-esterified fatty acid (NEFA; prepartum>0.3 mmol/L; postpartum>0.7 mmol/L, >1.0 mmol/L) and β-hydroxybutyrate (BHBA; prepartum>0.6 mmol/L; postpartum>1.0 mmol/L, >1.2 mmol/L) concentrations in smallholder (SH, n=15) and semi-commercial (SC, n=17) at 2 weeks prepartum and 1, 2, 4, and 8 weeks postpartum.

Group	Pre-parturition	Weeks after parturition							

		1		2		4		8	
				BHBA (mmol/L)					
	>0.6	>1.0	>1.2	>1.0	>1.2	>1.0	>1.2	>1.0	>1.2
SH (n=15)	46.7	33.3	26.7	33.3	33.3	40.0	26.7	20.0	13.3
SC (n=17)	52.9	41.2	29.4	41.2	41.2	35.3	29.4	47.1	35.3
SH+SC (n=32)	50.0	37.5	28.1	37.5	37.5	37.5	28.1	34.4	25.0

**Group**	**Pre-parturition**	**Weeks after parturition**							

		**1**		**2**		**4**		**8**	

				NEFA (mmol/L)					
	>0.3	>0.7	>1.0	>0.7	>1.0	>0.7	>1.0	>0.7	>1.0
SH (n=15)	86.7	73.3	40.0	60.0	26.7	40.0	26.7	53.3	6.7
SC (n=17)	58.8	17.7	11.8	17.7	11.8	11.8	5.9	0.0	0.0
SH+SC (n=32)	71.9	43.8	25.00	34.4	18.8	25.0	15.6	25.0	3.1

Patterns of dynamic in biochemical parameters and BCS changes also showed some differences between SH and SC. When considering based on loss of BCS, SC cows tended to have higher degree of NEB condition and lipolysis activity. Serum NEFA concentrations were higher in prepartum and greatly higher in SH compared to SC at postpartum period. The previous study showed reducing in energy intake at dry period resulted in early elevation in serum NEFA compared to adequate energy feeding cows [25]. In our study, it was possible that dairy cows in the SH group did not have proper nutritional management throughout late lactation to dry period; presumably cows were unable to gain BCS resulted in low BCS prepartum and higher in serum NEFA concentration and percentage of above baseline values in serum NEFA in the SH group.

After calving, peak of BHBA concentrations was also different between groups. The peak of serum BHBA concentrations was observed at 1 and 4 weeks postpartum in SC and SH, respectively. Difference in period of high serum BHBA concentrations could be resulted from different types of ketosis that was also reported in the previous study [26]. It was concluded that a failure of post-fresh cow nutritional management resulted in very high level of serum BHBA during 3-6 weeks postpartum, so called type I ketosis. Another type was type II ketosis, which was caused by failure of pre-fresh cow nutritional management, resulted in high level of serum BHBA during 1-2 weeks postpartum. Post-calving differences in energy balance of dairy cows also played an important role in NEB condition.

In postpartum period, SH might suffer from failure in nutritional management and other feed intake-depression disease resulted in type I ketosis. Normally in temperate areas, energy balance was negative during the first 3 weeks postpartum. Data showed that 7 weeks period of NEB in the tropics [27] resulted in longer elevation of serum BHBA in the SH group. Cows with NEB in SH did not show a strong reduction in BCS, which could be related to lower milk yields of this group compared to SC. On the other hand, cows in the SC group had higher BCS prepartum with peak of serum BHBA concentration at 1 week postpartum, which could be considered as type II ketosis. The reason for higher postpartum NEFA concentration but lower degree of BCS loss and serum BHBA concentration of SH compared to SC was still unclear and require further study. Difference in degree of elevation of serum NEFA and BHBA that resulted from metabolic adaptation of dairy cow was suggested in the previous study [28].

## Conclusion

It was possible that Thai tropical Holstein cows, which were developed for long period of time under humid tropical environment had ability to adapt metabolically to NEB condition. Moreover, from the adaptation point, dairy cows in SH tropical area might have more efficiency in utilization of ketone bodies as metabolic fuel, thereby, less BHBA concentration in serum. When cows exposed to long period of high serum NEFA and BHBA prepartum, it might have metabolic adaptations as same as a long-term ketogenic diet or intermittent fasting that has been observed in human. Therefore, study of liver TAG accumulation, factors related to ketone body synthesis and utilization should be required to provide complete information of homeostasis process related to NEB in SH tropical dairy farms.

## Authors’ Contributions

ST and TR designed the study. ST conducted the experiment and took care of all experimental animals. ST analyzed all samples. ST and TR performed the statistical analysis. ST drafted the manuscript. TR revised, edited, and finalized the manuscript. All authors read and approved the final manuscript.
